# Developing the practice context to enable more effective pain management with older people: an action research approach

**DOI:** 10.1186/1748-5908-6-9

**Published:** 2011-02-01

**Authors:** Donna Brown, Brendan G McCormack

**Affiliations:** 1 Acute/Chronic Pain Service, Second floor, West Wing, Royal Victoria Hospital, Belfast Health and Social Care Trust, Grosvenor Road, Belfast, Northern Ireland; 2 Institute of Nursing Research/School of Nursing, University of Ulster, Shore Road, Newtownabbey, Co. Antrim, Northern Ireland

## Abstract

**Background:**

This paper, which draws upon an Emancipatory Action Research (EAR) approach, unearths how the complexities of context influence the realities of nursing practice. While the intention of the project was to identify and change factors in the practice context that inhibit effective person-centred pain management practices with older people (65 years or older), reflective critical engagement with the findings identified that enhancing pain management practices with older people was dependent on cultural change in the unit as a whole.

**Methods:**

An EAR approach was utilised. The project was undertaken in a surgical unit that conducted complex abdominal surgery. Eighty-five percent (n = 48) of nursing staff participated in the two-year project (05/NIR02/107). Data were obtained through the use of facilitated critical reflection with nursing staff.

**Results:**

Three key themes (psychological safety, leadership, oppression) and four subthemes (power, horizontal violence, distorted perceptions, autonomy) were found to influence the way in which effective nursing practice was realised. Within the theme of 'context,' effective leadership and the creation of a psychologically safe environment were key elements in the enhancement of all aspects of nursing practice.

**Conclusions:**

Whilst other research has identified the importance of 'practice context' and models and frameworks are emerging to address this issue, the theme of 'psychological safety' has been given little attention in the knowledge translation/implementation literature. Within the principles of EAR, facilitated reflective sessions were found to create 'psychologically safe spaces' that supported practitioners to develop effective person-centred nursing practices in complex clinical environments.

## Background

Pain is one of the trigger reasons for people to seek healthcare assistance. However, evidence indicates that frequently the management of acute and chronic pain is inadequate [1,2]. Inadequate relief of acute pain increases the incidence and severity of postoperative complications and adverse outcomes, consequently increasing the cost of healthcare [1,3]. In a climate of cost-driven health services, many hospitals have in recent years achieved important improvements in postoperative pain management [4].

Older people offer distinct challenges, because pain not only lowers the individual's quality of life [5] but also predisposes them to a number of medical conditions, including; depression, sleep disturbances, anxiety, and occasionally aggressive behaviour [6,7]. Older people can be especially susceptible to identity threats (for example, dignity and respect [8,9], vulnerability [10], erosion of autonomy [11,12]) when they enter acute care [8,13]. In an environment that focuses on increased patient throughput, researchers argue that it is more difficult to care for older people as individuals [14,15].

Prior to pursuing the doctoral study reported on in this paper, a twelve-month in-depth ethnographic study was undertaken to explore issues relevant to older people in the acute hospital setting [13]. Patient interviews and observation of nursing practice revealed that holistic pain assessment with older people appeared deficient within the surgical environment, with nurses seemingly unaware of the importance of addressing the particular pain needs of older patients (Table 1). Data from the ethnographic study were subsequently fed back in writing to the study participants [13] and discussed in detail with nursing staff during two ward meetings. While nurses agreed with many of the findings, they articulated their frustration and concern that the research appeared to tell them what they were doing wrong, but failed to inform them how to change their practice. Having identified a starting point, they expressed an interest in understanding why they appeared to 'frequently get it wrong.'

**Table 1:** Outline of ethnographic study

Non-participant observation nursing practice (62 hours), patient interviews (n = 8), NWI-R questionnaire (Aiken and Patrician 2000):		
Revealed pain management practices with older people were deficient due to:		Ely's thematic analysis (1991) revealed three potential action cycles:
Limited/absent pain assessment.	}	Action cycle one: pain assessment and practice.
Inflexible analgesic prescriptions.		
Limited use of non-pharmacological strategies.	}	Action cycle two: Organisation of care.
Family and Physician opinion on use of analgesics.		
Fear of addiction.	}	Action cycle three: Knowledge and insight to deal with problematic pain.
Patients not being believed.		
Patients having decisions made 'for' rather than 'with' them.		

Contemporary literature on practice context suggests that it is a multi-layered construct that brings together issues of culture, leadership, behaviours, and relationships. In order to enhance effectiveness, multi-dimensional change strategies are required [16]. The importance of addressing cultural issues is well recognised in the knowledge translation literature. Drennan defined culture as 'the way things are done around here' [17]. Drennan's definition is derived from his studies of corporate culture, from which he concluded that culture is established from the habits, prevailing attitudes, and accepted behaviours of organisation members and therefore are manifested in how 'things are done around here' [17]. Although implementation of evidence-based practice and/or improvement in the quality of patient care is demanding [18], researchers should not be deterred from trying to change the culture and context in which practitioners work.

Researchers exploring evidence-based practice agree that context is an important but insufficiently understood mediator of change [19-24]. However, the complexity of context leaves it open to debate as to whether it can be measured by positivist [22,25,26] or more interpretative naturalistic approaches of inquiry [23,27-29]. The context in which nursing practice occurs is influenced by an infinite combination of boundaries and structures (such as staff relationships, power differentials, and organisational systems) that together shape the environment [24]. Therefore, theoretical models that have the potential to evaluate context in dynamic healthcare environments are necessary.

The Promoting Action on Research Implementation in Health Services (PARIHS) framework [30] has gained attention as a conceptual framework that may capture organisational influences on practice [22,27,31]. The authors of this work argue that three key elements -- evidence [32], context [33], and facilitation [34] -- should be considered when implementing evidence into practice. The element of context, within the PARIHS framework [30], is defined by the authors as 'the environment or setting in which the proposed change is to be implemented' [33], and this definition is used in the study reported in this paper. The subelements of context incorporate culture, leadership, and evaluation. Clarity concerning decision-making processes, patterns of power and authority, information and feedback mechanisms, and active management of competing priorities are all clearly defined boundaries within context. Often the nature of the environment or setting in which the proposed change is occurring is a key determinant of its success [35]. Thus, one of the major themes arising from context is culture, which manifests itself through the values, beliefs, and assumptions embedded within organisations [35]. Because there may be many cultures in any context, it is imperative to gain insight into the 'culture of a practice context,' if a sustainable approach to getting research into practice is to be achieved [33].

Previous research by the authors utilised the PARIHS framework set within an ethnographic methodology to explore practice context and gain an understanding of the factors that hindered effective pain management with older people [13]. The findings from this work are set out in Table 1. Although the ethnographic study identified contextual issues that needed to be addressed or changed, the methodology provided no opportunity to do so. Therefore, an additional research proposal (which formed the basis of the project reported on here) was developed to critically evaluate the findings from the ethnographic study and determine whether improved pain management practices could be achieved by working with practitioners in the unit to support a programme of change. This required an evaluation method that would address the issues in their entirety and concentrate upon creating and promoting a culture in which nurses recognized the need for improving their practice, sought knowledge and skills to do so, and felt supported, encouraged, and valued [36].

## Methods

Emancipatory Action Research (EAR) offers an approach that aims to improve practitioners' self-understandings and critique of their work settings [37]. Adopting a critical theoretical philosophy, this approach encourages participants to explore assumptions made in and about practice through systematic reflection and critique, making change the main interest of critical reflection [38]. Publishing the findings from this form of research is not without its difficulties: not least because the co-researchers are the main assessors of the effectiveness of the intervention, based on professional judgement, rather than external objective criteria [31]. EAR involves practitioner researchers in developing practice by introducing change in response to a need or problem [39]. This method was chosen because it enabled systematic working with ward-based practitioners to answer the research question: What effect would a programme of action research have on the practice of evidence-based pain management with older people following abdominal surgery?

### Objectives

The objectives of the study were:

1. To implement and evaluate a programme of development that enabled the team to critically analyse practice and put existing research into practice (evidence).

2. To develop effective teamworking to enhance pain management practices with older people (facilitation).

3. To develop an understanding of factors that inhibit or enhance pain management (context).

EAR best lends itself to the process of confronting unsatisfactory or distorted practices [37]. Within this form of research, facilitators assist practitioners toward enlightenment by fostering a culture of critical intent through reflective discussion [40]. It is a collaborative process that enables groups and individuals to develop and become empowered because it raises their consciousness of the influence they hold, and how to use their influence appropriately and recognise the aspects of decision making that are beyond their control [40].

The two-year project was undertaken in an abdominal surgical unit that consisted of two wards. Central to the study's success was the engagement of the lead nurse, ward managers (n = 2) and deputy ward managers (n = 2). These leaders along with eighty-five percent of nursing staff (n = 48) agreed, in writing, to participate; 11 senior registered nurses, 32 junior registered nurses, 5 healthcare support workers.

Adopting the principles of co-operative inquiry [41], all consenting nursing staff had the opportunity to work in focus groups (n = 5), facilitated reflective sessions (n = 18), *ad hoc *reflective sessions (n = 26), and consolidation workshops (n = 3) to explore their experiences and reflect together. The lead nurse and both ward managers also undertook to work individually with the lead researcher/facilitator (DB), using a model of 1:1 facilitation developed by Titchen [42] called 'critical companionship' (27 sessions in total). Critical companionship is described by Titchen [42] as a helping relationship in which one person accompanies another on an experiential learning journey. This shared learning can enable individuals and teams to transform practice cultures. It combines the processes of facilitating relationship building with the processes of critique, analysis, and evaluation of practice. It was anticipated that working within this framework, with the lead nurse and ward managers, at six weekly intervals, would enable greater self awareness, assist with finding solutions to challenging issues that arose from the project in a confidential, safe, and supportive environment, and offer an additional means of getting learning into practice.

Because healthcare settings are unpredictable, flexibility was essential to achieve community participation. Group work was negotiated monthly, in line with the nursing rota. This meant that any member of the nursing team who was on duty and had consented was able to participate. Consequently, membership within groups constantly fluctuated. To assist individuals and teams to understand the process and set the scene for all group work, ground-rules and a facilitation framework were formulated, verified, and adhered to throughout the project.

To address the objectives of the study and increase the accuracy and completeness of the data and outcomes, evaluation and affirmation of the data was achieved by:

1. Completing two episodes of non-participant observation of nursing practice (46 hours in total) midway and at the end of the project. Observation periods were negotiated with ward managers and staff one month in advance and conducted around the clock, in two hourly blocks. Field notes were systematically recorded on separate pages to record different types of data, including a page for observation of events (empirical) and difficulties or successes (method). At the end of each observation period, data were shared with the nursing team and reflective discussions were recorded (emerging themes). Finally, a personal notes page (reflexive notes) was maintained by DB.

2. Inviting six older patients to participate in pre and postoperative semi-structured interviews.

3. Completing the NWI-R Questionnaire [43] by 83% of registered nursing staff to provide further insight into the culture and nurse decision making in the unit.

### Focus groups

During focus groups, the ethnographic study findings [13] were discussed with participants in order to establish their credibility with them, *i.e*., if the data reflected their sense of reality. The data were then used to: provide a focus for discussion on the issues raised; examine nursing staffs' values and beliefs, through values clarification; and promote discussions within a claims, concerns, and issues framework [44]. Data were recorded using flip charts and verified at the conclusion of each meeting to ensure a collective understanding. Working in this way, it was possible to clearly identify the gap between the espoused values of person-centred practice and the reality of practice.

### Developing a vision for practice

Having completed five focus groups, nursing staff initiated a whole-team workshop with the aim of consolidating data gathered; developing shared values and beliefs; developing a shared language; and identifying action cycles and practical strategies for change. Ten members of the nursing team were able to participate. This included one ward manager, four senior registered nurses, three junior registered nurses, and two healthcare assistants (27% of overall consenting participants).

Creating a shared vision has been identified as an essential foundation stone in practice development [45,46]. Within the workshop, by examining the emerging themes and considering the issues within the context of the project, nursing staff developed a vision that was employed for the duration of the project and remained in place following its completion:

To develop efficient, high quality, holistic person-centred care in a dynamic environment where all patients, relatives and staff are equally respected and valued. We strive to develop teams where effective communication, education, and reflection are central to a supportive culture of developing practice

### Identifying action cycles

Having scrutinized the themes arising from the existing data with participants, it was decided that the three most pertinent issues requiring further work, in order to enhance pain management practices with older people, were:

1. Communication - action cycle one. Nursing staff agreed to explore ways in which they could improve communication throughout the multi-disciplinary team (MDT) as it impacted on all aspects of patient care, but was seen as particularly problematic for coping with episodes of severe pain.

2. Interruptions - action cycle two. Interruptions were considered a significant problem affecting pain management as well as other areas of practice. It was perceived that interruptions showed a lack of respect or understanding for nurses' work and patient care. Nurses sought ways in which they could reduce interruptions.

3. Pain assessment practices with older people - action cycle three. To improve pain assessment practices there was a need to identify key questions that all members of staff could use and increase knowledge for everyone on pain assessment.

To work on these action cycles, nursing staff chose to form small reflective groups that were entitled 'reflective sessions.'

### Facilitated reflective sessions

Reflection is fundamental to EAR, therefore facilitated reflective sessions became the key method for unravelling issues of context, defining and evaluating action cycles and developing, and refining strategic plans. Because we were working with emancipatory intent, reflective sessions held no preconceived agendas, only a clear understanding of the rules for engagement within the group and a determination to have a practical action plan, relating to an identified action cycle, at the conclusion of each session. To frame issues emerging within the practice context, ensure collective agreement and understanding and systematically map and assess how events unfolded or changed, qualitative data were recorded on flip charts, verified through group discussion at the end of each session, constantly reflected upon by participants, and scrutinized to identify possible themes arising using a staged approach as follows:

• Flip charts were used to record data as the groups discussed issues relating to their practice.

• At the conclusion of the reflective session, participants verified the data, assisted with drawing out the pertinent themes, and identified an action plan.

• Reflective notes with action plans were made available to the wider participating team through typed handouts.

• Diagrammatic representations of emerging themes where developed and placed on notice boards to encourage discussion and debate within the team.

• Workshops were organised to assess more widely how we were progressing and consider action taken and further work to be completed.

• Ely's (1991) thematic analysis was utilised to draw out themes with the nursing team.

• Individual reflective journals were maintained by four co-researchers.

Subsequently action plans were developed that facilitated team ownership and collective responsibility for changes in practice.

### Reflection and reflexivity as a guiding tool

Facilitating reflective practice in the turbulent and dynamic world of the acute hospital setting is not a comfortable or easy experience for those undertaking the journey. Confidence, flexibility and creativity are essential if people are to learn and remain willing to actively engage with the process [47]. Practitioners need to listen to themselves and others, so as to develop an understanding of their practice. However, this could only be developed through critical reflection, reflexivity, and dialogue [47].

Reflexivity can be defined as having an ongoing conversation about an experience while simultaneously living in the moment [48]. It encompasses a deep questioning of the mental, emotional, and value structures held by individuals/teams and their effect upon unfolding situations. To be reflexive, people have to stand back from values and belief systems, habitual ways of being, structures of understanding themselves, and their relationship with the world [38,47]. This requires generating an awareness of the way they are perceived and experienced by others, and being able to change deeply held ways of being [47].

As participants worked their way through the issues, DB was required to offer support by being generous of time, knowledgeable, and physically and emotionally 'present' [49]. Because this type of research is value-laden and inevitably political [50], DB's ability to be reflexive, deal with the issues as they unfolded, and be supportive to ward-based staff (at all levels) during the challenging times was fundamentally important. Therefore, throughout the project, DB maintained a reflexive journal and shared her reflections with her supervisor and a fellow doctoral student.

### Uncovering contextual issues and their impact on practice

A range of themes were identified demonstrating the complexity of contextual issues that impacted on effective person-centred practice (table 2). Data were analysed using Ely's (1991) [51] ten-step approach to data analysis:

**Table 2:** Items identified by the nursing team as impacting on person-centred pain management practices/patient care.

Elements of the PARIHS framework	Action cycles identified by ward nursing staff	**Themes arising from reflective strategies**.	**Themes merged through reflexivity and reflection on data**.
Evidence (1)	Communication (7)	Lack of support (10)	
Context (2) Sub elements Culture (3) Leadership (4) Evaluation (5)	Interruptions to nursing practice (8)	Value of nurses/nursing (11) Threat (12)	PSYCHOLOGICAL SAFETY
Facilitation (6)	Pain assessment practices (9)	Respect (13) Trust (14)	

		Time (15)	

		Oppression (16)	

		Power (17)	

		Distorted perceptions (18)	

		'Blame,' 'accusation' and 'criticism' (19)	HORIZONTAL VIOLENCE

		Autonomy (20)	

1. Study and re-study the raw data to develop detailed, intimate knowledge.

2. Note initial impressions.

3. List tentative subthemes.

4. Refine subthemes by examining the results of steps two and three, and returning to the entire database of step one.

5. Group data under the still tentative subthemes and revise subthemes if needed.

6. Select verbatim narrative to link the raw data to subthemes.

7. Study results of step 6 and revise if needed.

8. Identify themes and write theme statements based on the common characteristics of subthemes, and by linking data in and across subthemes.

9. Integrate findings of each data set.

10. Compare findings for commonalities or patterns, differences, and unique happenings.

Through this process, nursing staff discovered that their environment and subsequently pain assessment practices with older people were deficient due to: inadequate communication; multiple interruptions; insufficient understanding of the needs of older people; power imbalance (*e.g*., the dominant power of doctors); oppressive behaviours; horizontal violence; threat; a lack of autonomy; distorted perceptions; insufficient support, value, and trust (lack of psychological safety), time constraints; and weak leadership (Table 2).

**Figure 1:**
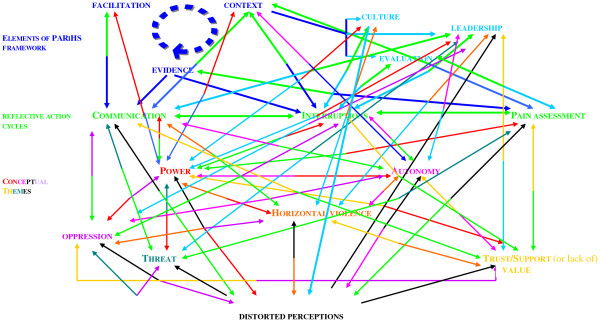
**Interconnected environmental issues uncovered that affected pain assessments practices with older people**.

Ongoing participatory analysis of the data revealed that the three action cycles (communication, interruptions, and pain assessment) were all interlinked and embedded in six overarching themes of context: leadership, psychological safety, oppressive behaviours, power and autonomy; horizontal violence; and distorted perceptions (Figure 1). These were judged to have a major effect on the ward environment. It became evident that we needed to address the overarching key issues arising from the practice context, whilst simultaneously paying attention to the three action cycles to effect any change in pain management practices with older people.

### Communication - action cycle one

Co-researchers deemed inadequate communication to be the overarching action cycle that was inextricably linked with issues of pain management, constant interruptions, and unreasonable demands of the wider MDT. They considered that inadequate communication led to a general lack of understanding and undervaluing of nurses' work. Nurses perceived they were criticised for their actions rather than being asked for their opinions; they considered that they had no power or autonomy and limited leadership or support to change the *status quo*. Consequently, this bred discontent and strained working relationships.

However, members of the MDT were not the only contributors to communication difficulties within the unit. Reflective sessions also exposed miscommunication that frequently occurred when nursing staff did not clearly state what assistance they required from one another. This ultimately fostered resentment when 'others' did not comprehend their needs. For example, senior nurses believed they were under 'the most pressure,' because they were required to complete tasks that junior nurses were not trained to do (*e.g*., change central line dressings, administer intravenous drugs). Although senior nurses were content to be asked to complete these tasks, because it was this that defined their seniority, they felt resentful because junior nurses did not necessarily complete tasks for them in return. When asked 'are you explicit in instructing junior staff?' the senior nurses realised that they did not direct junior nurses at these times; rather they expected them to know what was required. This resulted in nurses feeling devalued, increasing conflict in the unit and causing nurses to communicate their frustration by 'moaning to one another' or 'exploding,' due to the pressure of continued misunderstanding and miscommunication.

Five consecutive facilitated reflective sessions concentrated on the impact of working as undervalued people within the MDT. The action arising from these reflections was that nursing staff became more open with their instructions to one another.

### Interruptions - action cycle two

Nursing staff reflected upon how interruptions (*e.g*., people seeking information, telephone inquiries, being called away from their work with patients to attend to MDT colleagues requests for assistance) impacted on nurses' work and patient care. While they reasoned that interruptions were largely used as a form of communication, nursing staff found interruptions wearisome, particularly in circumstances where they compromised the patient's dignity. Interruptions were also considered to be a constant frustration at shift handover and medicine round times, because they were distracting for nurses and impacted negatively on patient care. Initially, nursing staff gave little consideration to how interruptions could be managed, because they were resigned to them being part of routine ward life and felt powerless to change this.

In an attempt to reduce the impact of interruptions, actions taken included freeing up a member of the nursing team to answer all queries at handover time, putting the patient first and asking members of the MDT to wait for a query to be answered, and role modelling behaviour by limiting interruptions among each other.

### Pain practices - action cycle three

Exploring issues of pain management revealed that nursing staff considered pain to be high on the older person's list of concerns and therefore a priority for them as nurses. Poor communication between patients, nurses, and doctors, insufficient time, ward pressures, constant interruptions, and unrealistic expectations of patients, families, and the organisation as a whole were cited as primary reasons for inadequate pain assessment practices. Older people were viewed as being 'silent sufferers' of pain, making it difficult for nursing staff to disentangle pain management from the ethos of care in general. Additionally, nurses saw the Acute Pain Team (APT) as being both an inhibitor and an enabler of their pain management practices. While they felt that the pain nurse specialists were knowledgeable, supportive, and approachable, equally they considered that the APT deskilled ward nurses, because they made decisions for them.

### Actions taken to address context issues

An overview of key themes, supporting excerpts, and action arising from facilitated critical reflection to alter the context in which nurses worked are displayed in Tables 3 and 4.

**Table 3:** Example of how action cycles, key themes, and excerpts relate to one another.

Themes	**Examples of issues unearthed during reflections with nursing staff**.	Post-project feedback
Communication Action cycle one	Ward Manager: 'Communication within the ward is deficient at times...we seem to repeat the same information.'	Ward Manager: 'I have learnt to be more professionally mature and communicate with MDT, as an adult.

Interruptions Action cycle two	A. Doctors (*e.g*., 'concurrent ward rounds,' 'doctors working one nurse off another to get what they want'). B. 'Multiple interruptions at handover time from other professionals.'	Ward manager: 'Interruptions are so difficult to manage.'

Pain assessment Action cycle three Older peoples' needs	Nurse: 'Older people don't tell you about their pain.' Support worker: 'You have to get a nurse to repeat what the doctor says, they don't seem to understand.'	Nurse: 'We discuss how we can improve practice and how we may better help older patients to understand their care.'

Power imbalance Horizontal violence	Nurse: 'I want the ground rules to say that there will be no recriminations for opinions....if someone doesn't agree with you, then they can't make your life difficult.'	Nurse: 'We discuss issues and how to move forward as a team.'

Value Support Trust Respect	Support Worker: 'It's like you don't exist until someone wants something.'	Nurse: 'Increased support has been invaluable.

Threat	Lead Nurse: 'It's frustrating when insufficient time is given for new initiatives to be established.'	Nurse: 'Things in the ward are generally better.'

Autonomy	Nurse: 'Why is it I'm allowed to make a decision to give a patient paracetamol today, but not tomorrow when the senior nurse is on duty?'	Nurse: 'It's better now we delegate and support each other.'

Distorted perceptions	Nurse: 'We are under more pressure than anyone else.' Ward manager: 'We always consult everyone about what we do.'	Nurse: Thinking things through with you (facilitator) permitted a more appropriate response and resulted seeing things differently.'

Leadership Support Value	Ward manager: 'I was avoiding conflict but now see that avoidance has led to an increase in issues.' Nurse: 'You need to know whose decisions count.'	Ward managers: 'I've developed insight into how important it is for me to be a strong leader.'

**Table 4:** Outcomes from the project gained through facilitated feedback and non-participant observation of nursing practice.

*Outcome*	*Action*	*Action cycle*
Non-participant observation of nursing practice revealed that nurses discussed pain with older patients when they were working with them.	Nursing staff use all available opportunities to speak to older people about their pain.	Communication Action cycle one
Nurse: 'We discuss how we can improve practice and how we may better help older patients to understand their care.'	Reflection revealed that many older people had impaired hearing. Action - nursing staff encouraged all members of the MDT to stand closer to older patients when they were speaking to them.	
Post research semi-structured interviews revealed that older people perceived that; 1. nursing staff assessed and treated their pain regularly, 2. they were partners in their care.		

Improved reflection skills	The nursing team introduced; - Reflection and feedback at the end of a shift for junior nurses who take charge.	Communication Action cycle one

Ward managers developed an understanding of the significance of role modelling behaviour.	- Attend the morning medical ward round to role model how it should be conducted and encouraging junior nurses to ask questions.	Communication Action cycle one
	- Take a patient caseload when the junior nurse is in charge of the unit to role model how to communicate with nurse in charge.	Interruptions Action cycle two

Senior ward nurses adopted a more facilitative approach to communicating with junior staff.	- Ask junior nurses guiding questions, rather than providing answers.	Communication Action cycle one

Ward nursing staff began to undertake new initiatives and evaluate these	- Incorporated changes into off duty gained through facilitated sessions.	
	- Setting target dates for implementing and evaluating changes, *e.g.*, discuss pain with older people when they are working with them.	Communication Action cycle one
		Pain assessment practices
	Completing a pain algorithm	Action cycle three

Drawing on the data to focus specifically on pain management practices with older people, one example of a change in practice is outlined below. This example elucidates how each action cycle impacted on another as ward staff attempted to enhance pain management in the unit.

Following a reflective session, one ward manager led on an action initiative to introduce an early morning medicine round. The nursing team reasoned that this change in practice would permit patients to receive analgesia prior to 'getting up and about' and allow nurses more freedom to attend the medical ward rounds to enhance MDT communication and reduce interruptions to patient care. Some nursing staff expressed concerns about giving analgesia to patients who were fasting prior to surgery, while others were reluctant to change from traditional practices. In response to these concerns, further reflection led to the nursing team completing an audit of medication adverse effects and the efficacy of the change being instigated. The results showed no increase in adverse effects and 92% of nursing staff considered that MDT communication had improved. Consequently, this change was permanently adopted. One nurse commented:

'The change to working patterns in the morning has had a positive effect as it permits us to spend more time with patients, because older people have analgesia on board, they can now do more for themselves.'

This change in the morning routine signified a major shift in the culture and mindset of the nursing staff working within the ward. The success with which they carried out this change encouraged nursing staff to engage with enthusiasm in the reflective process, enhanced nurse morale, and encouraged them to be innovative. Additionally, reflection assisted nursing staff to draw upon empirical evidence and their experience to develop a pain assessment algorithm.

### Insights developed into the complexity of practice context

The data from this study reveal new understanding of the complexity of practice contexts and the way these complexities impact on effectiveness in practice. Three characteristics of context were found to be the most significant in this study: power and autonomy, horizontal violence and oppressive behaviours, and leadership.

### Power and autonomy

Using facilitated sessions to unpick the themes with co-researchers/participants revealed that elements of power and autonomy were constantly at play. In nursing, clinical reality determines the socially constructed context that in turn affects clinical care [52]. Constraints and boundaries imposed within the clinical context mean that, for nursing staff, power retains an image of being something that is used to control and manipulate thoughts, attitudes, and social relationships. Nurses were uncomfortable discussing power, particularly when it was focused upon them [53], and they were challenged to consider strategies to shift ward culture. Issues of duty rosters, how staff valued each other's work, and alteration to ward routines proved contentious. Arguably this may have been because the nurses were predominantly women working in a patriarchal environment, thus linking power and oppression to one another. Lukes' [54] three dimensions of power may best elucidate issues of power exposed within the context of the unit. Within Lukes' model, one-dimensional power involves the capacity to directly influence events (*e.g*., the ability of a nurse or patient being directly involved in decisions concerning treatment). Two-dimensional power includes the ability to influence the agenda and prevent certain possibilities being considered (*e.g*., a senior nurse negatively influencing a junior nurses' decision making). Three-dimensional power involves the ability to control frameworks through which we make sense of and understand ourselves and the world (*e.g*., organisational and/or medical dominance over the working environment). The problem with this type of power is that it leads to individuals assuming that some issues are presupposed because an alternative cannot be seen or considered. For example:

Nurse one: 'Some days I am allowed to be in charge of my patients and make the decisions about their pain management, and on others days there is someone senior on duty and I need to be more careful, they are senior nurses and should make the decisions.'

Nurse two: 'You need to give people their place. They (senior nurses) are more confident and assertive.' (Focus group 2)

Nurse one: 'Sometimes I say the elderly patient is sore and needs something, but the senior nurse says I'm wrong.'

Facilitator 'Does he/she ask the patient?'

Nurse one: 'No they say I'm wrong?' (Reflective session 3)

Nurse one: 'I tried, at the start to change practice, but senior nurses do their own thing.... so what's the point in trying.' (Reflective session 6)

These extracts identify how some senior nursing staff can exert power over patients and junior nurses and effect optimal pain management practices. As a group of people who perceive a sense of powerlessness and helplessness, senior nurses may turn to oppressive behaviours that may be displayed in turning against those they consider as less powerful [55]. This potentially disempowers junior nurses and impacts upon the care older people receive as nurses see themselves as objects and powerless to influence some decisions. As we explored issues pertinent to pain management and older people, nursing staff aligned themselves with older people. They considered older people to be oppressed and silent and reasoned that this was similar to nurses and nursing; that is, their environment and subsequent behaviours were intertwined with issues of value and self worth, powerlessness, oppression, paternalism, and a sense of loss of control over their life resulting in dependency [56,57]. Consequently, power, like oppression, was seen to be insidious, serving the purpose of limiting an individual's freedom to choose. Having reflected upon these findings, nurses decided to value themselves, refrain from using statements such as 'I'm just the nurse,' and desist from avoiding the medical morning round.

### Horizontal violence and oppressive behaviours

Despite having aspirations of greater self value, as the project unfolded and nursing staff began to action the strategies developed through reflective sessions, predictably, a small number of senior nurses responded to the perceived threat to their identity by sabotaging any attempts to change practice. This manifested itself in devaluing others, criticism, gossiping (which exacerbated distorted perceptions), and negativity. All of these factors fall under the auspices of horizontal violence [58] and are associated with oppressive behaviours. Despite initial consensus for action being achieved, decisions began to be undermined and it became impossible to make initiatives work.

Constant undermining of initiatives [59] resulted in ward goals not being met. This increased levels of staff sickness, demoralised nursing staff and impacted negatively on patient care. As one ward manager struggled with the rising discontent and a feeling of isolation, she became unable to maintain effective leadership. The literature suggests that fear of punishment, being disliked, and isolated by nursing colleagues has the potential to prevent nurse managers from being assertive, which ultimately affects communication and how the manager is perceived [60]. Because the behaviour of nursing staff in this ward began to impact negatively on the ward environment, the nursing team, facilitated by DB, continued with weekly reflective sessions to work through the issues and honour agreed new ways of working. Simultaneously, the lead nurse, senior and deputy ward managers, and DB were challenged to examine what was occurring and what was required of them as leaders to transform the culture and context of the ward, utilising the critical companionship framework [42]. Through rational discourse [61] and consciousness raising [62], the nursing team developed insights into their situation and began to work together.

### Leadership

Leadership is seen to be a key issue in the way that a practice context is shaped [30,63,64]. How leaders perceive relationships within the team and the impact of these relationships on practice is critical to the way that an effective practice context is created [18]. Using the critical companionship model [42], Lucy (lead nurse), Daniel, and Sophie (ward managers) [pseudonyms] were individually encouraged to explore what they perceived were the challenges associated with being a leader. Discussions revealed that there were a number of common underlying issues in both wards (*e.g*., 'staff sickness and inadequate nurse numbers,' the influence of autocratic medical staff, nurses' inconsistent approach to their responsibility and accountability) that were perceived to influence practice to a greater or lesser extent. Reflection on leadership styles with the ward managers revealed that they primarily adopted a transactional approach to managing their individual wards.

Exploring the notion of transactional leadership and its potential effect on the context of the practice setting was demonstrated most clearly in the ward that experienced the greatest difficulty in changing the practice context. Over the course of the project Sophie, the ward manager, attempted to unfreeze [65] the core cognitive structures but experienced resistance to change from senior nurses. Consistent undermining of Sophie's authority left her isolated, unable to communicate effectively, and placed her in an untenable situation. Nevertheless, facilitated reflection offered Sophie the opportunity to identify, for herself, what the issues were and, although she was required to be courageous and open to challenge about her leadership style, she was able to move towards a transformational form of leadership (Tables 3 and 4).

Daniel also had a transactional approach to leadership. In particular, he had reservations about participating within the project because he was concerned it would threaten his authority. However, as he became fully immersed within the project he actively encouraged nursing staff to avail of the opportunity to reflect.

Because Daniel relinquished some of the power and control he had within the ward, nursing staff were enabled to identify initiatives to work upon, actioned them, and evaluated the outcome before moving to the next initiative. It is argued that working in this way offers the most successful means to secure a positive outcome [59,66].

Consequently, the team in this ward was able to gain consensus and work their way through the action cycles and strategies, which impacted positively on patient care. As they became more skilled in using reflection, nurses found themselves in a position to consider how they could enhance pain management practices with older people and developed a pain algorithm. Though the algorithm was not anything different from that which is available in the literature, notably they were able to produce it within a few weeks because it made sense to them within the context of their practice. Furthermore, towards the completion of the project, non-participant observation of nursing practice revealed that nursing staff where beginning to integrate the algorithm and reflection into their practice (*e.g*., a group of nurses asked DB to help them reflect after an older patient had experienced severe pain).

In contrast, the lead nurse (Lucy) had a transformational approach to leadership and the power to challenge the *status quo*. Participating in the project gave her insight into the issues arising from working with emancipatory intent. Having identified that there appeared to be a power struggle (in one ward) Lucy considered it was 'time to call some nurses to account.' This was something she had previously been reluctant to do, because she was concerned that it would suggest she was not working in a facilitative way.

Managers are charged with the responsibility of monitoring employee actions [67] to ensure results for patient care are achieved. However, one difficulty with transformational leadership is the misconception that leaders should be amiable to everyone [64]. Senior leaders are required to create an environment that encourages people to develop, motivate decision making, hold people accountable, and reward 'correct' behaviour [68]. It is imperative, therefore, that transformational leaders deal with issues appropriately, because this can make the difference between staff feeling empowered or abandoned [64]. The skill is knowing and balancing when to stand back and when to step in [66]. Critical companionship [42] helped Lucy to understand the need for leaders to challenge inadequate practice and call individuals or teams to account.

### The concept of 'presence' and its connection with psychological safety [59]

Practice is contextually located and embedded in multiple cultures that are created by actors in that culture [69]. Organisational culture has typically been described as the deeply engrained beliefs and values that frame actions and experiences in workplaces [17,70]. In acute healthcare organisations, individual ward cultures and ways of working can be highly distinctive. Bate [35] proposes that understanding organisational culture in the context of practice is key to understanding how best to bring about cultural change. Because many diverse and conflicting cultures may operate within the organisational context, perhaps the interconnected nature of culture and context may best be explained by drawing upon the analogy of a 'soup.' That is: expert chefs consider that stock is the essential secret ingredient of a well-made soup. While there are no hard and fast rules of how good stock should be made, there is general agreement that it should be prepared by simmering various core ingredients together. Simmering determines the intensity of flavour and encourages the impurities to rise, so that they might be skimmed off before the additional ingredients of the soup are added. Thus, the environment in which practitioners' work (organisational and ward context) becomes the stock of the soup. Appropriate facilitation and leadership (simmering) encourages practitioners to identify the culture and presenting issues (Tables 3 and 4). These subsequently represent the impurities that need to be altered (working as co-researchers) in order for the ward context and culture to be ready for action (that is, enhancing pain assessment and management).

Purpose, goals, and direction [66] are insufficient *per se *to alter the context in which practitioners work. 'Nothing undermines the creative process more than the naïve belief that once the vision is clear, it's just a matter of 'implementation' [49]. The strength and stability of culture is derived from the fact that it is group based, and if notions are deeply engrained, the group will resist change because they do not want to deviate from what they perceive is the norm [59]. Thus, culture is based upon shared learning experiences and taken for granted basic assumptions. All learning is about thinking and doing, how we (as individuals/teams) interact with the world, and the capacities we develop from our interactions [49]. Differences in learning lie with our depth of awareness and the source of our action.

Dewey's [71] learning cycle suggests that we learn from the past through cycles of reflection and action that subsequently result in new actions. However, the work of Senge *et al*. [49] may best assist with the theorising of the arising themes from this project. These authors propose that there is a second type of learning, in which we learn from the future and in discovering our role in bringing that future into being. In a society that is experiencing profound change, Senge *et al*. [49] are unconvinced that learning based on the past remains an adequate guide to the future. They propose that when demanding and complex issues require in-depth understanding, commitment, and sustained change, a different process is necessary. Senge *et al*. [49] therefore present the image of a 'U.' The authors contend that the 'U' extends what happens in the learning process by distinguishing different levels of perceiving reality and action that follows from it. The three levels proposed are sensing, presencing, and realising. Sensing incorporates gathering information to gain insight into that which is occurring (*e.g*., through the ethnographic study and focus groups with nursing staff). Presencing is the deep reflection stage, where individuals or groups try to reach a state of clarity and complete connection with what is occurring, to a state of 'inner knowing' (understanding), (*e.g*., working through the issues in Sophie's ward). Realising is the action phase where individuals or teams bring something new into reality (*e.g*., addressing difficult issues, developing an algorithm). The depth of sensing and presencing holds the key to the success of realising [49].

Arguably, presencing builds on Mezirow's [72] concept of perspective transformation, because it requires people at all levels of an organisation to surrender their perceived need for control and stand back to observe what is occurring. Senge *et al*. [49], propose that the 'U' can assist with developing a language in which people can talk and think together. Movement down the 'U' results in the clear progression and transforming of our habitual ways of seeing. Alternatively, movement up the 'U' signifies transforming the source of our awareness. It is the bottom of the 'U,' where presencing occurs (requiring people to retreat, reflect, and allow inner knowing to emerge, thus transforming ones self and will to 'let go'). This element of the 'U' however, remains relatively unexplained and is identified as the area in which people experience difficulty in shifting their view. Within presencing, a deep source of seeing and connection to what is emerging makes decision making obvious. Thus, operating at the bottom of the 'U' is where realisation of what is needed occurs, followed by the desire to act accordingly.

The lead nurse, ward managers, and DB were required to work at the bottom of the 'U' as difficult issues unfolded. The dilemma was that time was needed for deeper reflection and learning to occur, but the pressure and anxiety created from challenging situations demanded urgent action. Senge *et al*. [49] define prototyping as a way of accessing and aligning new insights by bringing the understanding of our head, heart and hands together. Effective prototyping requires acting on an issue before it is complete or perfect, and learning to listen to feedback to develop helpful clues about how to proceed. While shifting our awareness of our world and ourselves is anxiety provoking, making us want to return to former ways of being, this is not always possible because we have become conscious of the limitations of our traditional way of being. Thus, within the project as DB, Lucy, and Sophie became immersed within presencing and tried to move towards prototyping, there was a need for DB to unconditionally support individuals/teams, to step outside of events, and consider possible options.

**Figure 2:**
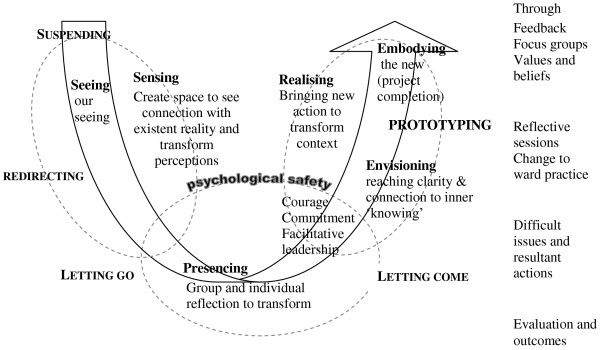
**Capacities of the U movement in relation to the project (adapted from Senge *et al*. 2005, p219) **[49].

Senge *et al*. [49] argue that we need to use our intuition and emotions, rather than objective analytical rationalism, if we are to unlock the future we seek through presencing. Hence, it is argued that the data arising from this project fit with and possibly shed light on what may occur at the bottom of the 'U' (Figure 2). Working closely with nursing staff in the surgical unit offered the opportunity to reflect and raise consciousness of habitual ways of seeing things (Tables 3 and 4). Reflective sessions, critical companionship, and reflexivity revealed a whole range of underlying interlinked and interconnected issues (Figure 1) that needed to be addressed before changes to pain management practices with older people could begin. This process was, at times, emotional and difficult for those who participated in the project. As nursing staff unpicked the issues and reflected upon them, often they were uncomfortable with where it was taking them; nevertheless, they needed to explore the issues fully if they were to transform the self, let go and identify meaningful actions.

Working at the bottom of the 'U' also required the development of a psychologically safe environment [58], consistent, strong, facilitative leadership and encouragement to work through the issues, especially when teams and individuals met obstacles when working through action plans. The essence of psychological safety is to create an environment where people feel able to focus on underlying issues without threat of loss of self-identity or integrity. Schein [59] suggests that motivation to learn new ways of thinking or behaving can be repressed if learning anxiety and discouragement becomes overwhelming. Senge *et al*. [49] further argue that discouragement and fear prevent us from changing the systems in which we are embedded. Where tensions, such as those experienced with changing context exist, trust becomes eroded (through people experiencing disappointment, fear, and anxiety), people feel vulnerable and may decide to refrain from engaging or cooperating. In this instance, the option for avoiding or exiting from the project and the critical companionship relationship [42] became a real possibility for the lead nurse. Reverting to habitual ways of being constitutes reactive learning, which is governed by 'downloading' habitual ways of thinking and continuing to see the world within the familiar categories we are comfortable with. Possibly these are the times when trust, support, and safety (of self and others) become most closely interconnected and require the use of the whole self to ensure containment of the unfolding situation. Ultimately, the learner must come to realise that a new way of being is possible and achievable [59].

## Summary

Initially, practitioners and DB embarked on a journey to explore the effect a programme of action research would have upon enhancing pain management practices with older people. Utilising the principles of EAR, facilitated reflective sessions were found to create 'psychologically safe spaces' that supported practitioners to develop effective person-centred nursing practices in complex clinical environments.

Reflective critical engagement with the findings revealed that context is a dynamic, complex, and somewhat anarchic phenomenon, with many issues blending together to create a 'soup' of factors that enable or inhibit effective nursing practice. 'Ward culture' impacted not only on pain management practices, but also influenced all aspects of ward life and patient care. Therefore, it is probable that the theme of pain management practices with older people could be substituted with other areas of speciality nursing practice (for example, tissue viability) to achieve enhanced patient outcomes.

Many studies have examined the practice context [13,22-26], however it continues to be the case that few studies have explored, in-depth, the experience of addressing the complex elements of practice context in order to positively affect the practice culture. Whilst other research has identified the importance of 'practice context' and models and frameworks are emerging to address this issue, the theme of 'psychological safety' has been given little attention in the knowledge translation/implementation literature. It is argued that the unobservable unique elements of context require methodical consideration and exploration if they are to be adjusted in positive and sustainable ways, just as with a soup whose flavour needs to be adjusted to meet individual tastes.

Because the quality of this form of research cannot be assured by the rigorous application of predetermined strategies or procedures [39], readers are required to consider if the findings resonate with their experience [73]. If the findings are meaningful and applicable to their individual experiences, then this project meets the criterion of fittingness.

## Competing interests

The authors declare that they have no competing interests.

## Authors' contributions

DB presented the original work as a poster (KU07) and presentation (KU08) at an annual Knowledge Utilization colloquium, prepared and conducted the majority of the proposal, research, and analysis of findings. DB and BGMcC prepared and wrote the original grant proposal. BGMcC provided critical feedback and contributed to amending and refining the paper. DB and BGMcC read and approved the final manuscript.
